# Tissular perfusion influence on central, mixed and atrial venous oxygen saturations

**DOI:** 10.1186/cc12672

**Published:** 2013-06-19

**Authors:** SH Goto, BF Mazza, FR Machado

**Affiliations:** 1 Hospital São Paulo, Universidade Federal de São Paulo, SP, Brazil; 2 University of São Paulo, Universidade Federal de São Paulo, SP, Brazil

## Introduction

Even though there has been quite a discussion on whether venous oxygen saturations are useful to guide treatment during initial resuscitation of sepsis, using mixed and central venous oxygen saturations as goals is still advised in the Surviving Sepsis Campaign under strong recommendation but a low level of evidence (1C). According to these guidelines, SvO_2 _<65% or SvcO_2 _<70% demands treatment. In addition, there is no consensus whether these variables are interchangeable. The objective of this study was to evaluate the influence of tissular perfusion on the correlation between the central venous (SvcO_2_), the mixed venous (SvO_2_) and the atrial oxygen saturations (SvaO_2_) by the analysis of arterial lactate.

## Methods

A prospective observational study; the populations from three ICUs of the Hospital São Paulo were evaluated from October 2011 to November 2012 and patients diagnosed with severe sepsis or septic shock monitored by pulmonary artery catheter (PAC) were included. Hyperlactatemia was defined as an arterial lactate value >28 mg/dl and the correct location of the PAC was confirmed by chest radiography and pulmonary artery pressure tracings. For the statistical analysis, samples were allocated into two groups: normal lactate levels (Group 1) and hyperlactatemia (Group 2). Results were expressed in mean ± standard deviation or median (25 to 75% percentiles) or percentages.

## Results

**Figure 1:**
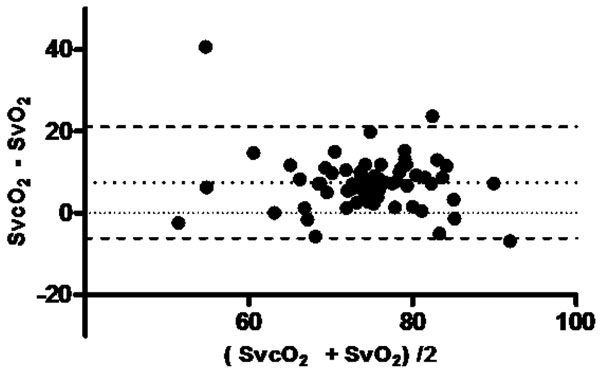
**Bland-Altman (SvO_2_×SvcO_2_): bias 7**.52, bias SD 6.957. 95% Limits of agreement: -6.116 to 21.16.

**Figure 2:**
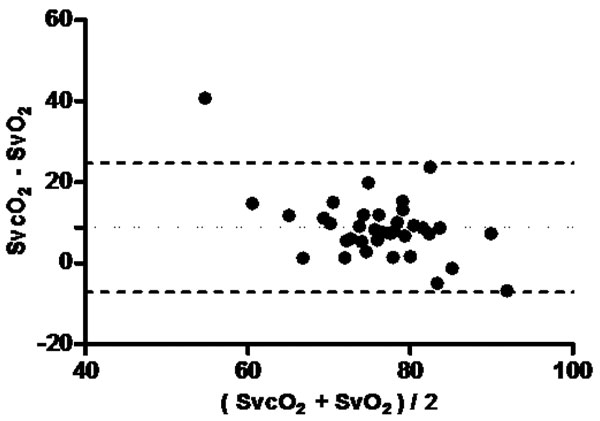
**Bland-Altman (SvO_2_×SvcO_2_) in Group 1: bias 8**.778, bias SD 8.137. 95% Limits of agreement: -7.171 to 24.73.

**Figure 3:**
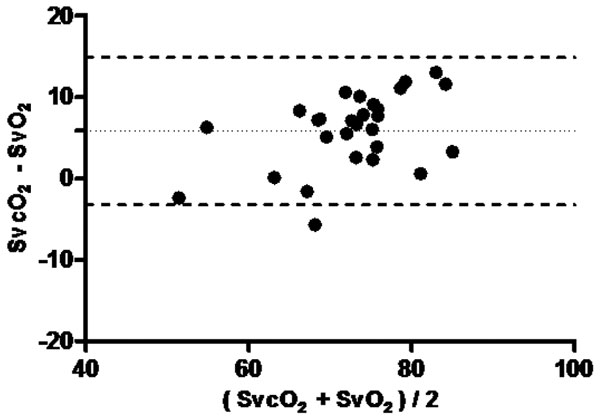
**Bland-Altman (SvO_2_×SvcO_2_) in Group 2: bias 5**.857, bias SD 4.627. 95% Limits of agreement: -3.211 to 14.93.

Twenty-one patients were included; altogether, 65 paired blood samples were obtained (Table 1). A higher correlation between the venous oxygen saturations was found in the hyperlactatemia group (Table 2). APACHE II and SOFA scores were higher among these individuals (Table 1). SvcO_2 _and SvO_2 _were shown not to be acceptable surrogates by the analysis of the Bland-Altman plots, but bias and limits of agreement were narrower in Group 1 (Figures 1, 2 and 3).

**Table 1:** Baseline

Variable	Group 1 (*n *= 37)	Group 2 (*n *= 28)	*P *value
Age	66 (54 to 69)	65 (54 to 69)	0.171
Male (%)	35.1 (13)	85.7 (24)	0.000
APACHE II	18 (17 to 22)	24 (20 to 29)	0.016
SOFA admission	6 (5 to 10)	10.5 (7 to 12.75)	0.007
SOFA sample	9 (7 to 11)	11 (9.25 to 16)	0.007
SvO_2 _(%)	72 (68.5 to 76.5)	70.5 (67 to 73)	0.182
SvcO_2 _(%)	81 (76 to 85)	77 (72 to 80)	0.016
SvaO_2 _(%)	77.9 ± 8.6	74.3 ± 9.7	0.274
SaO_2 _(%)	98 (97 to 98.5)	95 (93 to 96)	0.000

Results expressed as % (*n*) or mean ± standard deviation or median (25 to 75% percentiles). APACHE, Acute Physiologic Chronic Health Evaluation; SOFA, Sequential Organ Failure Assessment.

**Table 2:** Spearman correlation (*r*)

Variable	Total (*n *= 65)	Group 1 (*n *= 37)	Group 2 (*n *= 28)
SvO_2_×SvcO_2_	0.74*	0.66*	0.83*
SvO_2_×SvaO_2_	0.68*	0.60*	0.82*
SvcO_2_×SvaO_2_	0.72*	0.63*	0.85*

*P <0.05.

## Conclusion

In patients with hyperlactatemia, a global tissular perfusion marker, venous oxygen saturations presented a higher correlation and narrower bias and limits of agreement, suggesting, perhaps, that under high arterial lactate levels there is a generalized hypoperfusion that reflects not only on the SvO_2_, but also on the SvcO_2. _There was no agreement between those variables either.
